# Supplementary material for: Mechanisms governing the pioneering and redistribution capabilities of the non-classical pioneer PU.1
Source: Nat Commun. 2020 Jan 21;11:402. doi: 10.1038/s41467-019-13960-2 (PMC6972792; doi:10.1038/s41467-019-13960-2)
Supplement: Supplementary file 7 — Source data [file 41467_2019_13960_MOESM7_ESM.zip › Source_Data/FigureS2/FigureS2B_MotifScanOutput/KmeansCluster7/homerResults/motif50.similar.html]

motif50

## Information for motif50

A
C
G
T
A
G
T
C
G
A
C
T
G
T
A
C
A
C
G
T
G
A
T
C
A
G
C
T
A
G
T
C
A
G
T
C
A
C
G
T
T
A
C
G
A
G
T
C
A
G
C
T
A
T
C
G
  
Reverse Opposite:  

A
T
G
C
C
T
G
A
C
T
A
G
A
T
G
C
C
G
T
A
A
C
T
G
A
C
T
G
C
T
G
A
C
A
T
G
C
G
T
A
A
C
T
G
C
G
T
A
T
A
C
G
G
T
C
A
  

|  |  |
| --- | --- |
| p-value: | 1e-22 |
| log p-value: | -5.125e+01 |
| Information Content per bp: | 1.872 |
| Number of Target Sequences with motif | 27.0 |
| Percentage of Target Sequences with motif | 0.84% |
| Number of Background Sequences with motif | 12.5 |
| Percentage of Background Sequences with motif | 0.03% |
| Average Position of motif in Targets | 108.5 +/- 31.7bp |
| Average Position of motif in Background | 142.3 +/- 51.8bp |
| Strand Bias (log2 ratio + to - strand density) | 0.8 |
| Multiplicity (# of sites on avg that occur together) | 1.04 |
| Motif File: | file (matrix) reverse opposite |

### Similar de novo motifs found

|  |  |  |  |  |  |  |  |
| --- | --- | --- | --- | --- | --- | --- | --- |
| Rank | Match Score | Redundant Motif | P-value | log P-value | % of Targets | % of Background | Motif file |
| 1 | 0.959 | C T G A C T A G A T G C C T G A C A T G T C A G C G T A C T A G C T G A A T C G C G T A T C A G T G C A | 1e-20 | -46.237922 | 1.56% | 0.25% | motif file (matrix) |
| 2 | 0.814 | A G T C A G C T A G T C A G C T A G T C A G T C A C G T A C T G A G T C | 1e-12 | -29.684722 | 4.17% | 2.00% | motif file (matrix) |
| 3 | 0.825 | C G T A A C T G A G T C C G T A A C T G A C T G G T A C A C T G C G T A A C T G G T A C A C T G | 1e-12 | -27.902112 | 0.53% | 0.03% | motif file (matrix) |
